# Breastfeeding Practices, Demographic Variables, and Their Association with Morbidities in Children

**DOI:** 10.1155/2015/892825

**Published:** 2015-08-10

**Authors:** Dipen V. Patel, Satvik C. Bansal, Archana S. Nimbalkar, Ajay G. Phatak, Somashekhar M. Nimbalkar, Rajendra G. Desai

**Affiliations:** ^1^Department of Pediatrics, Pramukhswami Medical College, Karamsad, Gujarat 388325, India; ^2^Department of Physiology, Pramukhswami Medical College, Karamsad, Gujarat 388325, India; ^3^Central Research Services, Charutar Arogya Mandal, Karamsad, Gujarat 388325, India

## Abstract

Appropriate feeding practices are the key contributor to reducing morbidities and mortalities in under-five children. A cross-sectional questionnaire based survey of mothers of children aged less than 5years was conducted in 781 mothers. More than half of mothers (57.5%) started feeding within an hour of birth, 55.9% gave exclusive breastfeeding for six months, 89.1% of the mothers stopped breastfeeding before two years of age, 18.2% of the mothers bottle-fed the babies, and 15.6% had problems during breastfeeding in first 6 months. Early initiation of breastfeeding within one hour of birth promoted exclusive breastfeeding, and breastfeeding for longer duration. Exclusive breastfeeding increased frequency of feeds. Multivariable logistic regression showed that initiation of breastfeeding after an hour of birth (*p* = 0.035), not providing exclusive breastfeeding for 6 months (*p* < 0.0001), unemployed mothers (*p* = 0.035), having two or more kids (*p* = 0.001), and complementary feeds given by person other than mother (*p* = 0.007) increased hospitalization. Starting breastfeeding after an hour of birth (*p* = 0.045), severe malnutrition (*p* = 0.018), and breastfeeding for < two years (*p* = 0.026) increased rates of diarrhea. Breastfeeding practices were not optimum and interventions to improve these practices need to be strengthened.

## 1. Introduction

Appropriate Infant and Young Child Feeding (IYCF) practices are essential for optimal growth, cognitive development, and overall well-being in early vulnerable years of life. Malnutrition contributes to about 60% of under-five mortality worldwide annually and over two-thirds of these are due to inappropriate feeding practices [1]. An analysis showed that appropriate breastfeeding and complementary feeding practices can alone prevent under-five deaths by 19% [2].

The World Health Organization (WHO) recommends exclusive breastfeeding for the first six months of life and the addition of complementary feeds from six months onwards, with continued breastfeeds till at least two years of age [3, 4].

Apart from exclusive breastfeeding initially, time of introduction, content, and consistency of complementary feeds are critical for early nutrition. The early introduction of complementary feeds before the age of six months can lead to displacement of breast milk and increased risk of infections, besides the babies being physiologically immature. Similarly, inadequate and inappropriate complementary feeding with unhygienic practices leads to recurrent and persistent infections and malnutrition which is followed by growth retardation, immunodeficiency, and eventually fatal outcomes. This is a concern for Indian scenario, where previous studies have suggested inability to maintain exclusive breastfeeding and late introduction of complementary feeds [5–7]. Evidence based studies have stressed the importance of human milk and concluded that infant feeding should be considered as basic health issue rather than lifestyle choice [8].

Breastfeeding though is a natural act; it is a behavior that needs to be learned. Mothers and other caregivers need active assistance for optimum breastfeeding practices. The Global Strategy for Infant and Young Child Feeding describes the essential interventions to promote, protect, and support exclusive breastfeeding [9].

Multiple efforts are being envisaged to improve feeding practices in children. WHO and United Nations Children's Fund (UNICEF) launched the Baby-Friendly Hospital Initiative in 1992, Integrated Management of Childhood Illness (IMCI) in mid-1990s, and IYCF in 2002 which stress the importance of breastfeeding. All these programs have been adopted by India since the last one to two decades to promote appropriate feeding practices in children under-five years.

Appropriate breastfeeding and complementary feeding practices depend on accurate information and support from the family, community, and healthcare system. Inadequate knowledge about feeding practices is an equally important determinant of malnutrition, as is the lack of adequate and hygienic food. However, in spite of all the efforts deployed as information, education, or training campaigns, the prevalence of proper feeding practices remains low [10].

India hosts more than one-third of the world's children who are wasted. From under-five children in India, 43% are underweight and 48% are stunted due to chronic undernutrition. Only 25% of newborns were put to the breast within one hour of birth and 46% are exclusively breastfed [10].

Multiple factors are responsible for this slow improvement in nutritional health indicators in India. According to World Bank report, India's Integrated Child Development Services (ICDS) needs to undergo significant changes to address the current malnutrition crisis in India [11].

As there is a paucity of literature on the feeding practices in this region, the present study was undertaken to assess the feeding practices of mothers having children under-five years of age. The interaction of sociodemographic variables with feeding practices and associations of childhood morbidity were studied.

## 2. Materials and Methods

### 2.1. Study Design

This study is designed as a questionnaire based survey.

### 2.2. Study Site and Population

This study was conducted at the pediatrics outpatient department (OPD) catering to children from birth to 18 years of age attached to a tertiary care Shree Krishna Hospital (SKH) of Pramukhswami Medical College located at Karamsad from Anand district of Gujarat, India, over a period of four months from October 2013 to January 2014. The hospital caters to Anand and Kheda districts of Gujarat. Mothers having children less than five years of age attending the clinic for immunizations, growth monitoring, treatment of minor illnesses, health check-ups, follow-up, and so forth were interviewed after taking an informed consent. All children with known nonnutritional congenital or acquired reasons (e.g., congenital heart diseases, cerebral palsy, genetic disorders, and tuberculosis) of failure to thrive were excluded.

### 2.3. Study Procedure

The questionnaire consisted of 39 questions on sociodemographic variables, feeding practices, and morbidities of children (hospitalization, diarrhea, and acute respiratory illnesses). Weight of the child was taken on a digital weighing scale of Zeal Medical Pvt. Ltd. Weight for age classification was used to estimate the nutritional status of children using the *z*-score cut-off point of WHO Global Database on Child Growth and Malnutrition [12]. Nutritional status was defined with *z*-score of <−3 *z* as severe undernutrition, between −3 and −2 as moderate undernutrition, between −2 and +2 *z* as normal, and >+2 as overweight. The questionnaire was in English but the interviews were conducted in local language (Gujarati/Hindi/English) as per mothers' convenience. The OPD runs between 9:00 a.m. and 1:30 p.m. and between 3:00 p.m. and 5:00 p.m. Interviews were conducted between 10 a.m. and 1:00 p.m. and each interview took approximate 15 to 25 minutes to finish. Data was entered on a predesigned, structured questionnaire on Magpie (Datadyne) application on a Generation 2 iPAD running on iOS6 and later uploaded on the server website on daily basis. The services provided by the website were free of charge. Data was transcribed to Microsoft Office Excel 2010 and then imported to SPSS 14 (SPSS Inc., Chicago, IL, USA) for analysis after validation and cleaning.

### 2.4. Statistical Analysis

Descriptive statistics were used to depict the sociodemographic characteristics, clinical profile, and feeding practices of the study population. Associations between feeding practices and sociodemographic variables as well as between feeding practices, sociodemographic variables, and hospital admissions at univariate level were determined using chi-square test. Multivariable logistic regression was performed to determine independent associations. Odds ratio (OR) was calculated and significance was considered at *p* value < 0.05.

### 2.5. Ethical Considerations

The study was approved by human research ethics committee of HM Patel Centre for Medical Care and Education, Karamsad.

## 3. Results

Out of 6385 children who visited pediatrics OPD between October 2013 and January 2014, 4416 (69.16%) were below 5 years of age. As the interviews were conducted between 10:00 h and 13:00 h, a total of 910 children could be approached. Out of these, 811 were accompanied by their mothers. Twenty-four mothers did not give consent due to time constraints while 6 were not eligible because of the known cause of failure to thrive (i.e., 3 had congenital heart diseases, 2 had cerebral palsy, and 1 had pulmonary tuberculosis). Thus 781 (97%) eligible mothers participated in the study.

The mean (SD) age of participating mothers was 27.18 (3.77) years (range: 19 to 42). Majority of mothers (86.1%) had studied up to 10th standard. Very few (2.6%) mothers had multifetal pregnancy. The monthly family income was quite skewed ranging from Rs. 2000 to Rs. 45000. Majority of the children belong to age group of six months to two years (54.8%) and had an older sibling (55.5%). The mean (SD) birth weight of the children was 2708.94 (510.92) grams (range: 900 to 4500). Most of them suffered from diarrhea (61.7%) and cough and cold (77.6%) and required hospitalization (41.9%) (Table 1).

As per WHO definition of nutritional status as per weight for age [12], 411 (55.5%) were normal, 78 (10.5%) had moderate undernutrition, 149 (20.1%) were severely malnourished, and 102 (13.8%) were overweight. Total duration of breastfeeding more than 1 year (*p* = 0.002) and exclusive breastfeeding for 6 months were associated with lower rates of severe malnutrition (*p* = 0.003).

More than half of the mothers (57.5%) started breastfeeding within one hour of child's birth, gave exclusive breastfeeding for recommended 6 months (55.9%), and continued to breastfeed in the second year of life (50.7%). Most mothers did not face any problem during breastfeeding in the first six months (84.4%) and did not bottle-feed the baby (81.6%). More than half of the mothers washed their hands (72.1%) before giving feeds and washed utensils after each feed (76.3%). Only 26.5% of the mothers who gave complementary feeds stored the food in oven or refrigerator and 42.2% always rewarmed the stored food before giving; however, 61.9% of the mothers often gave freshly prepared food. The complementary feed was given mostly by the mother (86.6%) (Table 1).

Primiparous mothers and mothers with multifetal pregnancy started breastfeeding early. Mothers who breastfed their children within 1st hour of birth were more likely to give exclusive breastfeeding for 6 months, breastfeed for a longer duration, and engage in bottle-feeding the baby. Maternal education was not associated with time of starting breastfeeding. Mothers who exclusively breastfed their children for recommended period of 6 months gave breastfeeding for longer duration and frequently breastfed their child in a day (Tables 2 and 3).

Multivariable logistic model revealed that initiation of breastfeeding within one hour, exclusive breastfeeding for six months, maternal employment, primipara status, mother feeding the child, and overweight significantly reduced hospital admissions. The predictive value of the model was good with 65.3% correct classification rate (Table 4).

Severe malnutrition, not starting breastfeeding within 1st hour of birth, and stopping breastfeeding before 2 years were associated with increased diarrhea episodes. The predictive value of the model was good with 67.5% correct classification rate (Table 5).

Children of unemployed mothers and having severe malnutrition were more likely to encounter cough/cold episodes. Hygiene of hands and utensils showed inverse association with cough/cold episodes. The predictive value of the model was good with 79.5% correct classification rate (Table 5).

## 4. Discussion

Early initiation of breastfeeding and exclusive breastfeeding of children below six months are considered the most decisive indicators for assessing breastfeeding practices [13].

In the present study 57.5% of the children received breastfeeding within one hour of birth. Similarly a study done at Allahabad showed 55.8% of the mothers initiating breastfeeding within six hours [14]. However, the National Family Health Survey (NFHS-3) 2005-2006 estimates are lower for India and Gujarat being 23.5% and 25.4%, respectively [15]. Another recent Indian study from Madhya Pradesh recorded similar low 26% mothers initiating breastfeeding in first hour [16]. The higher percentage found in our study can be attributed to the fact that the NFHS was done about 9 years ago and was based on community based survey. Discrepancies from other Indian studies can be due to regional and cultural differences. Delayed initiation of breastfeeding has been recognized as a risk factor for neonatal mortality and according to a previous study, an estimated 22% of neonatal deaths could be prevented if breastfeeding was started within the first hour after birth [17]. Even though there is an increase compared to the NFHS data, the current rate is still inadequate to achieve the estimated mortality benefits of early initiation.

Exclusive breastfeeding during the first 6 months and therefore timely introduction of complementary feeding have many proven advantages to both the mother and the child and are therefore the prime focus in infant feeding promotional activities. In our study we observed 55.9% of the infants exclusively breastfeed till complete six months. Similar rates were observed by Das et al. (58.7%) and Banapurmath et al. (60%) [18, 19]. Indian studies by Kulkarni et al. (70.2%) and Nimbalkar et al. (85.6%) recorded much higher rates [20, 21]. Last NFHS-3 data of India and Gujarat shows 46.3% and 47.8% of infants in 0–5 months of age exclusively breastfed. And from the 6th month onwards 55.8% and 57.7% of infants received complementary feeding in India and Gujarat, respectively. Conversely, other Indian studies from Delhi showed lower recordings with 16.5% and 17.5% mothers starting timely complementary feeding [5, 6]. Appropriate duration of exclusive breastfeeding with timely introduction of complementary feeds is essential for growth and well-being of the child. An estimated 10% to 15% of under-five deaths in resource poor countries could be prevented if 90% of babies were exclusively breastfed for the first 6 months [2]. On multivariate analysis we found that children not exclusively breastfed for 6 months and started on breastfeeding after one hour of birth had significantly higher hospital admissions.

The NFHS-3 data for Gujarat state reports 93.5% of mothers breastfeeding between 6 and 9 months, which is similar to our data of 93.3% after 6 months. However, in the second year the breastfeeding rate observed in our study (50.7%) was lower than seen in NFHS-3 data (76.8%).

The rate of bottle-feeding was 18.4% similar to what was observed in NFHS-3 and in other studies. It is one of the optional indicators of IYCF practices. Bottle-feeding should be discouraged—bottles are difficult to clean and sterilize and are the source of infection, and artificial teats cause “nipple confusion” thus interfering with breastfeeding. The bottle-feeding was 11% in a study from urban population [22]. We found higher rates of bottle-feeding in children who were breastfed within 1st hour of birth.

Maternal illiteracy has been associated with suboptimal feeding practices [23]. We did not observe associations between maternal education with duration and initiation of breastfeeding on univariate analysis. Maternal education plays a huge role in increasing the receptivity of mothers towards correct practices. Studies from India have suggested significant association of maternal literacy and timely initiation of complementary feeding [5, 24, 25]. Lower literacy in mothers, in addition to lack of knowledge about correct practices and recommendations, makes routine counseling by community health workers also ineffective [26].

We did not find significant association of maternal occupation with initiation of breastfeeding and duration of exclusive breastfeeding despite housewives supposedly having more time available to feed their infants. One of the probable reasons for this is that working mothers carry their children at workplace and they are able to provide breastfeeding. A study in Malaysia reported that facility at workplace allowing mothers a flexible time to express breast milk helps in maintaining lactation [27]. Conversely, there have been other studies observing maternal occupation associated with suboptimal feeding practices [28–31]. On multivariate analysis, we found higher hospitalizations for children of housewives.

In our study we found an association of multifetal births with initiation of breastfeeding within one hour of birth and no relation with duration of exclusive breastfeeding. However, small sample size in multiple pregnancies (*N* = 16) was a limitation. Studies from Japan recorded significantly lower rate of exclusive breastfeeding and shorter duration of breastfeeding among twins or triplets than among singleton babies [32, 33]. Multiple births are seen as a hindrance to breastfeeding because of frequent association of prematurity with multiple pregnancies with lack/weakness of sucking reflex [34]. However in a study done in Canada on term newborns (singleton and twin), mothers of twins were again found less likely to exclusively breastfeed (adjusted odds ratio (OR) 0.30, 95% confidence interval (CI) 0.25–0.36) [35]. Prenatal education and counselling regarding feeding practices, such as alternating breasts, increases the initiation and duration of breastfeeding [3, 36]. Supporting and educating the families with birth of multiple babies increased breastfeeding by 74% [34].

In our study primiparous mothers initiated breastfeeding earlier than multiparous mothers. Microbial contamination of feeds is a major cause of infections leading to higher hospital admissions. A study from Madhya Pradesh showed significant association of hygienic practices and malnutrition in infants [16]. Meager data on hygiene practices during food preparation and feeding are available. Results of a study in north western Nigeria showed that only 28% of mothers washed their hands before preparing foods [37]. A study from slums of Dhaka showed 33% of the mothers exclusively breastfeeding while 54% of the nonexclusively breastfeeding mothers did not clean their child's hands before feeding [38]. Similarly another study from India showed just 17% hand cleaning before feeding [24]. We found higher values on hygienic practices like hand washing of mothers and washing utensils after each feed, but it was not up to the mark. On multivariate analysis we found reduced episodes of cough and cold in children whose mothers washed their hands and utensils sometimes as against the reference category of always washing hands and utensils, respectively. These findings may be because of varied culture, hygienic habits, and home environment, which were not evaluated in detail in the current study.

We found an association between hospital admissions of children with feeding given by person other than mother. Study from Maharashtra showed feeding given by foster mothers (elder brother/sister) as a major determinant of faulty feeding practices [39]. Mothers having two or more kids and severe malnutrition in children were associated with higher rates of hospital admission while overweight was actually protecting against hospital admissions.

We conclude that there has been slight improvement in feeding practices since the last NFHS survey in 2005-2006. However, the current rates of proper feeding practices are still well below the guidelines for Infant and Young Child Feeding. Late initiation of breastfeeding, nonexclusive breastfeeding, bottle-feeding, maternal illiteracy, and unhygienic feeding practices are still prevailing. Though more emphasis in terms of antenatal counseling, hospital delivery, and training of grass root workers on IYCF is being conducted to improve child feeding practices, they are still inadequate to improve short term and long term outcomes of childhood nutrition. There is an alarming need of a prospective educational intervention study or new innovative practices to achieve the optimum feeding practices in children under the age of five years.

## Figures and Tables

**Table 1:** Sociodemographic variables.

Sociodemographic variable	Frequency
Age (years) of the mothers, mean (SD)	27.18 (3.77), range: 19 to 42 years
Mother's education less than 4th standard	109 of 780 (13.97%)
Low birth weight children	195 of 768 (25.4%)
Monthly family income less than Rs. 15000	570 of 765 (74.5%)
Multiparous mothers	431 of 777 (55.5%)
Number of children who required hospitalization	327 of 781 (41.9%)
Number of children who had diarrhea	482 of 781 (61.7%)
Number of children who had cough-cold	606 of 780 (77.6%)
Multifetal pregnancy	17 of 778 (2.2%)
Number of mothers who had problems during breastfeeding during first 6 months after delivery	120 of 771 (15.6%)
Family support for breastfeeding	76 of 766 (10%)
Male gender	476 of 780 (61%)
Bottle feeding	142 of 771 (18.4%)
Employed women	138 of 779 (17.7%)
Hindu religion	653 of 779 (83.8%)
Started breastfeeding within 1st hour of delivery	443 of 771 (57.5%)
Age of the child (of total 767)	
Less than 6 months	60 (7.8%)
6 months to 24 months	420 (54.8%)
More than 24 months	287 (37.4%)
Use of soap to wash hands for children more than 6 months before feeding	404 of 687 (58.8%)
Washing utensils after each feed	532 of 697 (76.3%)
Washing hand of mother before each feed for children more than 6 months age	499 of 692 (72.1%)
Who feeds the child more than 6 months	
Mother	600 (86.3%)
Other person	95 (13.7%)
Exclusive breastfeeding for 6 months in children more than 2 years	160 of 286 (55.9%)
Breastfeeding stopped less than 2 years of age in children more than 2 years	253 of 284 (89.1%)

**Table 2:** Univariate analysis for time of starting breastfeeding after birth in children more than 2 years.

Variables	When did breastfeeding start?		*p* value
	After 1st hour of birth *N* (%)	Within 1st hour of birth *N* (%)	
Elder siblings			
No	129 (37.7)	213 (62.3)	0.018
More than/equal to 1	197 (46.2)	229 (53.8)	
Births			
Multifetal	2 (12.5)	14 (87.5)	0.014
Single	325 (43.2)	428 (56.8)	
Studied up to			
Less than 4th standard	49 (45.4)	59 (54.6)	0.51
4th standard and above	278 (42)	384 (58)	
Bottle feeding^*∗*^			
No	283 (86.8)	346 (79.0)	0.003
Yes	43 (13.2)	92 (21.0)	
Duration of breastfeeding^*∗*^			
Less than 2 years	293 (97.1)	371 (93.5)	0.032
2 years or more	9 (2.9)	26 (6.5)	
Exclusive breastfeeding^*∗*^			
Other than 6 months/more than 1 year	161 (49.1)	171 (38.8)	0.004
6 months	167 (50.9)	270 (61.2)	

^*∗*^Column percentages are reported.

**Table 3:** Univariate analysis for exclusive breastfeeding in children more than 2 years.

Variables	Duration of exclusive breastfeeding		*p* value
	Other than 6 months *N* (%)	6 months *N* (%)	
Problems in breastfeeding in first 6 months			
No	101 (42.1)	139 (57.9)	0.095
Yes	25 (55.6)	20 (44.4)	
Studied up to			
Less than 4th standard	18 (46.2)	21 (53.8)	0.776
4th standard and above	108 (43.7)	139 (56.3)	
Bottle-feeding			
No	96 (42.9)	128 (57.1)	0.438
Yes	30 (48.4)	32 (51.6)	
Occupation			
Employed	22 (47.8)	24 (52.2)	0.574
Housewife	104 (43.3)	136 (56.7)	
Sex of child			
Female	56 (50.5)	55 (49.5)	0.083
Male	70 (40.0)	105 (60.0)	
Religion			
Christian	7 (50.0)	7 (50.0)	0.498
Hindu	111 (44.9)	136 (55.1)	
Muslin	8 (33.3)	16 (66.7)	
Nutrition			
Normal	65 (40.4)	96 (59.6)	0.581
Moderate malnutrition	21 (45.7)	25 (54.3)	
Overweight	1 (50.0)	1 (50.0)	
Severe malnutrition	35 (50.0)	35 (50.0)	
Duration of breastfeeding^*∗*^			
Less than 6 Months	47 (14.7)	0 (0.0)	<0.0001
6 to 23 months	260 (81.2)	361 (94.2)	
24 months or more	13 (4.1)	22 (5.8)	
Number of breastfeeds per 24 hours^*∗*^			
Less than 8 times	270 (80.4)	312 (71.5)	0.005
8 times or more	66 (19.6)	124 (28.5)	

^*∗*^Column percentages are reported.

**Table 4:** Variables associated with hospital admissions in children aged more than 6 months.

Variables	*p* value	OR	95% CI for OR	
			Lower	Upper
Starting of breastfeeding				
After an hour of birth	0.035	1.482	1.027	2.138
Within hour (reference category)				
Exclusive breastfeeding				
6 months (reference category)				
Less than 6 months or one year and more than 1 year	<0.0001	2.100	1.450	3.039
Occupation				
Employed	0.035	0.585	0.356	0.963
Housewife (reference category)				
Elder siblings				
One and more	0.001	1.923	1.326	2.788
No (reference category)				
Feeding given by				
Mostly mother (reference category)				
Other	0.007	2.070	1.219	3.515
Nutritional status of children	0.006			
Moderate malnutrition	0.939	1.024	0.562	1.866
Overweight	0.011	0.441	0.233	0.831
Severe malnutrition	0.052	1.578	0.996	2.499
Normal (reference category)				

**Table 5:** Variables associated with diarrhea and cough/cold episodes in children age more than 6 months.

Variables	*p* value	OR	95% CI for OR	
			Lower	Upper
Variables associated with diarrhea in children aged more than 6 months				
Starting of breastfeeding				
After an hour of birth	0.045	1.479	1.009	2.166
Within hour (reference category)				
Nutritional status of children	0.029			
Moderate malnutrition	0.758	0.909	0.497	1.666
Overweight	0.209	0.695	0.394	1.227
Severe malnutrition	0.018	1.853	1.111	3.089
Normal (reference category)				
Duration of breastfeeding				
Up to 2 years	0.026	2.506	1.114	5.638
2 years or more (reference category)				

Variables associated with cough and cold in children aged more than 6 months				
Hand washing before each feed				
Always (reference category)				
Sometimes	0.039	0.620	0.394	0.977
Nutritional status of child	0.115			
Moderate malnutrition	0.590	1.221	0.590	2.527
Overweight	0.636	1.182	0.591	2.363
Severe malnutrition	0.015	2.135	1.159	3.933
Normal (reference category)				
Washing utensils after each feed				
After each feed (reference category)				
Sometimes	0.001	0.457	0.282	0.740
Occupation				
Employed	0.022	0.546	0.325	0.916
Housewife (reference category)				
